# Scheduling optimization based on particle swarm optimization algorithm in emergency management of long-distance natural gas pipelines

**DOI:** 10.1371/journal.pone.0317737

**Published:** 2025-02-10

**Authors:** Huichao Guo, Runhua Huang, Shuqin Cheng

**Affiliations:** 1 School of Safety Science and Emergency Management, Wuhan University of Technology, Wuhan, Hubei, China; 2 Zhejiang Business College, Hangzhou, Zhejiang, China; 3 Columbia University School of International and Public Affairs, New York City, New York, United States of America; Aalto University, FINLAND

## Abstract

This paper aims to solve the scheduling optimization problem in the emergency management of long-distance natural gas pipelines, with the goal of minimizing the total scheduling time. To this end, the objective function of the minimum total scheduling time is established, and the relevant constraints are set. A scheduling optimization model based on the particle swarm optimization (PSO) algorithm is proposed. In view of the high-dimensional complexity and local optimal problems, the neighborhood adaptive constrained fractional particle swarm optimization (NACFPSO) algorithm is used to solve it. The experimental results show that compared with the traditional particle swarm optimization algorithm, NACFPSO performs well in both convergence speed and scheduling time, with an average convergence speed of 81.17 iterations and an average scheduling time of 200.00 minutes; while the average convergence speed of the particle swarm optimization algorithm is 82.17 iterations and an average scheduling time of 207.49 minutes. In addition, with the increase of pipeline complexity, NACFPSO can still maintain its advantages in convergence speed and scheduling time, especially in scheduling time, which further verifies the optimization effect of the algorithm in emergency management.

## 1. Introduction

In natural gas emergency scheduling management, the most critical measurement indicator is the duration of scheduling, which directly determines the immediacy and efficiency of emergency response. If prompt measures are not taken, it may lead to far-reaching safety and economic threats. Unfortunately, traditional emergency scheduling methods for natural gas often encounter problems of poor control and regulation, especially insufficient attention to effective resource utilization, excessive reliance on tasks, and time constraints. Faced with these challenges, this article considers integrating particle swarm optimization (PSO)-based scheduling strategies into natural gas emergency management scheduling. The application of PSO algorithm has significantly improved the management effectiveness of natural gas scheduling in emergency situations.

PSO algorithm, as an intelligent optimization algorithm, has obvious advantages in all kinds of optimization problems because of its simple operation, powerful global search ability and easy implementation. However, the traditional PSO algorithm is prone to local optimization and slow convergence when dealing with high-dimensional and multi-constraint problems. In order to overcome these shortcomings, this article further applies the neighborhood adaptive constraint fractional particle swarm optimization (NAFPSO) algorithm. By dynamically adjusting the neighborhood topology and applying the penalty function constraint processing technology, the diversity and convergence speed of the algorithm are significantly improved. Compared with the existing methods, NAFPSO algorithm can better balance local search and global search in the optimization scheduling process, so as to ensure the effectiveness of the scheduling scheme and significantly improve the scheduling efficiency.

In this article, a natural gas pipeline emergency management scheduling optimization model based on NAFPSO algorithm is established, and the objective function and constraint conditions are defined. The advantages of the algorithm in convergence speed and scheduling time are verified by simulation experiments. The performance is analyzed deeply, and some suggestions for improvement are put forward. The main contribution is to propose a NAFPSO algorithm suitable for long-distance natural gas pipeline scheduling, verify its effectiveness and feasibility, and demonstrate its superiority compared with other optimization algorithms through comparative experiments, providing practical guidance and theoretical basis for future pipeline scheduling decisions. In terms of the structure of the article, the first chapter is the introduction; the second chapter is the literature review; then, the method and experimental design are elaborated; finally, the research results and contributions are summarized, and the future research direction is prospected.

## 2. Related work

In addition, designing an emergency response mechanism for natural gas transmission pipeline accidents [1–4] is a method to compress scheduling time in this regard. When an emergency safety accident occurs in a natural gas transmission pipeline [5], relevant departments can handle the accident according to the corresponding emergency measures in the mechanism. This method can indeed respond quickly to long-distance natural gas pipeline accidents, but this mechanism lacks basic information data [6]. The emergency rescue scheme lacks effective operability and reliable verification, so there is a lack of data and technical experience support in actual operation. Li Xiaolong [7] et al. designed an emergency GIS module to address the lack of GIS (Geographic Information System) [8] support and insufficient utilization of spatial information in traditional long-distance natural gas pipeline emergency management systems, and integrated it into the long-distance natural gas pipeline emergency management system. Through technologies such as map query and spatial analysis, functions such as emergency resource and linkage department positioning, accident impact analysis, and rescue path planning have been achieved, providing spatial information support for emergency schemes and assisting decision-making and command. Compared to the Yang Meng’s scheme, this scheme solved problems such as validation, operability, and information data, but it performed poorly in minimizing scheduling time.

More and more scholars have studied the application and generalization ability of PSO in different fields. In order to improve cloud computing load prediction, both Bacanin N [9] and Predić B [10] optimized network parameters by modifying the particle swarm optimization (PSO) algorithm. More accurate load prediction was realized. The results showed that the proposed method was superior to the traditional method in forecasting accuracy, and could deal with nonlinear and dynamic load characteristics effectively. Esfandyari M [11] combined machine learning and PSO algorithms to optimize ultrasonic excited two-tube heat exchangers. The results showed that the optimized system performed well in terms of heat transfer efficiency and energy consumption, which significantly improved the performance of the equipment. Moazen H [12] proposed the PSO-ELPM (Particle Swarm Optimization-Extended Lagrange Multiplier Method) algorithm, which combined elite learning, enhanced parameter updating, and exponential mutation operator improvement. Experiments showed that PSO-ELPM showed faster convergence rate and better solution quality on multiple test functions, which was especially suitable for dynamic and complex optimization environments. Bacanin N [13] studied an energy-efficient offloading mechanism using particle swarm optimization in 5G edge nodes. By optimizing data offloading strategies, energy efficiency was significantly improved and latency was reduced, improving the overall performance of 5G networks. In terms of scheduling, another extension of the PSO algorithm proposed in this article is the Hybrid PSO Algorithm [14], as well as the Multi-objective particle swarm optimization (MOPSO) [15], Parallel Particle Swarm Optimization (PPSO) [16], and Improved Particle Swarm Optimization (IPSO) [17]. These algorithms also have considerable advantages in scheduling optimization, as they can fully consider the complexity of scheduling networks [18] and multi-point to multi-point scheduling. However, they are not applicable to the emergency management of long-distance natural gas pipelines in this article, as the performance of these extended PSO algorithms is usually greatly affected by parameter settings [19], including the number of particles, maximum iteration times, inertia weights, etc. In practical applications, parameter settings may need to be repeatedly adjusted and optimized [20] to achieve better scheduling optimization results, which leads to the scheduling time being repeatedly extended and missing the optimal pipeline maintenance target time [21].

Therefore, this article used the NACFPSO algorithm to optimize the emergency management scheduling of long-distance natural gas pipelines. This algorithm not only considered the complexity of scheduling and global search ability, but also rationalized parameter settings to minimize the impact of parameters on the accuracy of the algorithm. When setting the objective function, it was weighted and the sum of scheduling time was refined, considering only the two most important constraints of time window and resources. Even without expanding the constraint range, it did not affect the control of scheduling time. Finally, a set of scheduling experiments for long-distance natural gas pipeline faults were simulated. The results showed that the NACFPSO algorithm greatly adjusted the scheduling time of long-distance natural gas pipelines, improved resource utilization, reduced emergency response time, and improved response efficiency, bringing significant improvements to emergency management of natural gas scheduling and enhancing the overall management level.

## 3. Physical model

### 3.1. Problem application

Based on the characteristics of long-distance natural gas pipeline emergency management, this article takes multi-point to multi-point, multi-constraint long-distance natural gas pipeline emergency management scheduling as the optimization problem. When facing emergency management of long-distance natural gas pipeline systems, there is a complex scheduling problem, which is how to quickly and effectively allocate maintenance resources in the event of sudden failures in the pipeline system, in order to minimize scheduling time and reduce potential risks. This issue involves multiple considerations: firstly, it is necessary to consider the structure and parameters of the pipeline system [22], such as the length, diameter, flow rate, etc., of the pipeline. Secondly, it is necessary to consider the availability of maintenance resources [23], including maintenance personnel, vehicles, and maintenance equipment.

### 3.2. Objective function

The formula for the minimum total scheduling time is:


$$\begin{array}{l} \text{Minimize }f\left(T_{\text{Scheduling}}\right)=w_{1}\cdot T_{\text{detection}}+w_{2}\cdot T_{\text{response}}+w_{3}\cdot T_{\text{maintenance}}+w_{4}\cdot \\ T_{\text{communication }} \end{array}$$ (1)

**Fig 1:**
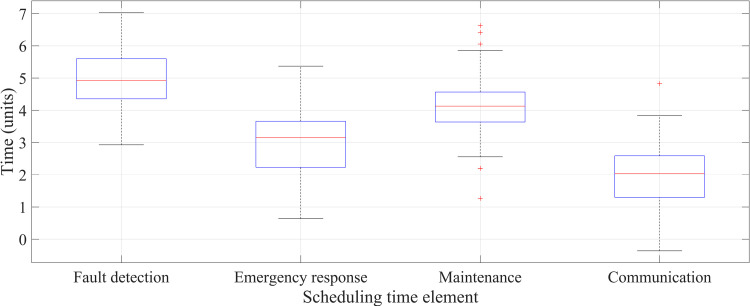
Objective function box diagram.

In the formula, $T_{\text{detection}}$ represents the fault detection time; $T_{\text{response}}$ represents emergency response time; $T_{\text{maintenance}}$ represents maintenance time; $T_{\text{communication}}$ represents communication time. In emergency management, effective communication is the key to ensuring coordinated action among all parties. ${\text{w}}_{1}$, ${\text{w}}_{2}$, ${\text{w}}_{3}$ and ${\text{w}}_{4}$ refer to the weight coefficients respectively. The box diagram of the objective function is shown in Fig 1.

Fig 1 shows the distribution of four scheduling time components: fault detection time, emergency response time, maintenance time, and communication time. Through observation, the change range of fault detection time is large, indicating its instability, while the change of communication time is minimal, indicating that its stability is high. These changes reflect the importance and reliability of each time component in scheduling and help optimize scheduling strategies.

### 3.3. Constraints

#### Resource limitations.

The requirement for setting the number of available maintenance personnel is $\sum_{i=1}^{N}x_{i}\leq C_{\text{maintenance personnel}}$the requirement for setting the number of available vehicles is $\sum_{j=1}^{M}\;y_{j}\;C_{\text{vehicles}}$the requirement for setting the number of available maintenanceequipment is $\sum_{k=1}^{P}\;z_{k}\leq \;C_{\text{maintenance equipment}}$

*N*, *M* and P all stand for quantity. The terms $x_{i}$$y_{j}$and $z_{k}$ respectively represent the total number of maintenance teams, vehicles, and equipment used. The responsibility of the maintenance team is to provide immediate maintenance and disposal in case of pipeline problems or other emergency situations. It is abundant in quantity and can ensure immediate and efficient intervention for all faults, ensuring stable operation of the pipeline system at all times. Similarly, the professional knowledge and literacy of maintenance personnel also have a direct impact on the efficiency and quality of emergency handling. The main function of this vehicle is to transport repair personnel, equipment, and various materials to the fault location, and in emergency situations, it also undertakes the tasks of evacuation and material transportation. Adequate and diverse traffic flow not only ensures effective execution of emergency situations, but also achieves high flexibility and speed in emergency response. Maintenance equipment is a comprehensive network composed of various tools, equipment, and instruments, mainly used for fault detection, repair, and replacement work. Having sufficient maintenance tools and types can ensure the disorder of the maintenance process and enhance the efficiency and likelihood of success in fault response. $C_{\text{maintenance personnel}}$ $C_{\text{vehicles}}$ and $C_{\text{maintenance equipment}}$ respectively represent limitations on the number of available maintenance personnel, vehicles, and maintenance equipment.

#### Time window.

Fault detection time window:


$$T_{w}=\frac{2\cdot L\cdot A}{\rho \cdot V}+\sum_{i=1}^{n}\frac{L_{i}}{V_{i}}+\frac{D^{2}\cdot \rho }{f\cdot \mu }$$ (2)

Emergency response time window:


$$T_{MTW}=\frac{\left(\sum_{i=1}^{n}\;\theta_{i}\cdot G_{i}\right)+\kappa +\left(\sum_{j=1}^{m}\;\lambda_{j}\cdot H_{j}\right)+\mu }{\nu }$$ (3)

Repair time window:


$$T_{ETW}=\frac{\left(\sum_{i=1}^{n}\;\alpha_{i}\cdot D_{i}\right)+\beta +\left(\sum_{j=1}^{m}\;\gamma_{j}\cdot F_{j}\right)+\delta }{\epsilon }$$ (4)

In the formulas, $T_{w,\;}T_{MTW,\;}T_{ETW}$ respectively represent the time required for fault detection, response time in emergency situations, and time required for repair. L refers to a specific length of the fault. A  refers to the area involved. ρ refers to the density parameter. ${\text{V}}_{\text{i}}$ refers to the speed of detection of the fault point. μ indicates the fault detection parameter. ${\text{θ}}_{\text{i}}$ refers to the response time parameter. ${\text{G}}_{\text{i}}$ refers to the response requirements associated with the point of failure. ${\text{H}}_{\text{j}}$ refers to vehicle-related processing requirements. ${\text{λ}}_{\text{j}}$ refers to the responsiveness parameter. ${\text{α}}_{\text{i}}$ refers to the repair parameter. ${\text{D}}_{\text{i}}$ is for complexity. ${\text{F}}_{\text{j}}$ refers to the repair requirements related to the point of failure. β and δ refer to the extra time associated with the repair process. *ϵ* refers to the error term. The fault detection time describes the time required from the occurrence of the fault to the detection and confirmation of the fault. Emergency response time refers to the time required for pipeline operators to quickly activate emergency schemes to ensure the safe operation of pipelines in the event of sudden disasters or emergencies. Through accurate and timely fault diagnosis, pipeline operators can quickly grasp the working status of pipeline systems, identify abnormal situations in a timely manner, and implement necessary preventive measures to avoid further deterioration of accidents. Repair time refers to the minimum time required for emergency repair when a malfunction is detected. With the expansion of urban long-distance natural gas pipeline networks and the increasing demand for gas quality from users, it is crucial to ensure that gas transmission facilities can be quickly and accurately repaired and the normal operation of pipeline systems can be restored in the event of emergencies, reducing the interruption time of faults and mitigating the adverse impact on gas supply. This is crucial for meeting the daily gas demand of users.

#### Safety requirements.

The safety distance requirement is set to $D_{\text{safety}}\geq D_{\text{minimum safe distance}}$The definition of safety distance is the safety distance that must be maintained around the pipeline to prevent environmental hazards from causing damage to the pipeline or affecting its normal operation. The basis for determining the safe distance is the nature of the pipeline, surrounding environmental conditions, potential hazards, and other comprehensive considerations. The minimum safe distance refers to the shortest distance that must be kept away from pipelines to ensure personal and property safety in the event of an accident. The minimum safe distance is usually determined based on factors such as the operating parameters of the pipeline, the types of accidents that may occur, and the degree of impact when accidents occur. $D_{\text{safety}}$ indicates the safe distance; $D_{\text{minimum safe distance}}$ represents the requirement for the minimum safe distance.

#### Service level.

The requirement for setting the emergency response time is ${\text{T}}_{\text{response}}\leq {\text{T}}_{\text{target}}$The requirement for setting the repair time is ${\text{T}}_{\text{maintenance}}\leq {\text{T}}_{\text{target}}$. ${\text{T}}_{\text{target}}$ represents the desired service level target time. Establishing, monitoring, and evaluating target timelines can continuously optimize and improve emergency management capabilities. It is necessary to timely summarize experiences and lessons learned, improve emergency schemes and processes, and enhance the ability of organizations and personnel to respond to emergencies. It is also necessary to compel relevant departments and personnel to pay attention to emergency response, accelerate troubleshooting and repair, and improve their ability to respond to emergencies.

## 4. PSO and NAFPSO

### 4.1. PSO algorithm

**Fig 2:**
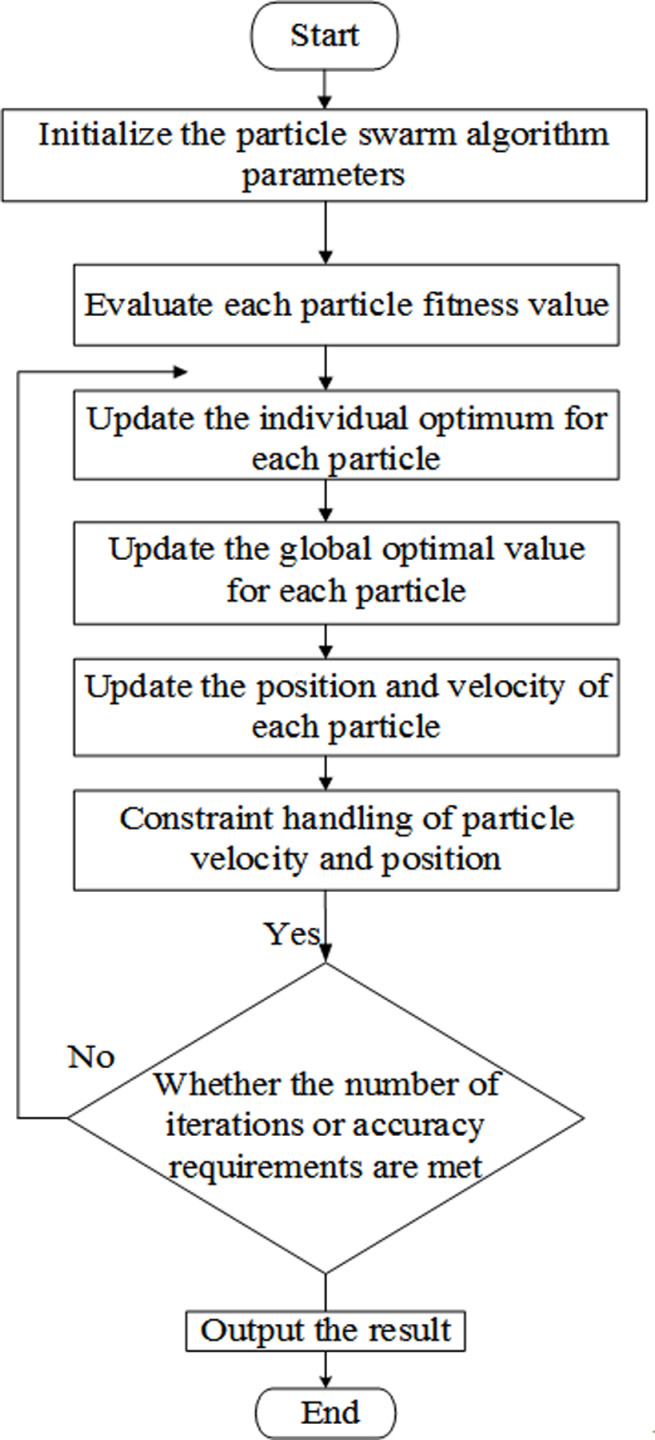
PSO algorithm flowchart.


In the 1990s, inspired by the foraging behavior of bird flocks, the PSO algorithm was proposed. PSO consists of NP n-dimensional individuals $x_{i}=$$\left\{x_{i,1},x_{i,2},\cdots ,x_{i,n}\right\}\left(i=1,2,\cdots ,NP\right)$ iteratively searching for the optimal solution in the feasible domain. Among them, NP is the population size, and n is the dimension, usually taken as the number of decision variables in the optimization problem. During the iteration process, each individual updates their current velocity and position by learning from their own historical and population optimal positions. The velocity and position update formulas for individual *i* are shown in Formulas (5) and (6). The implementation process of PSO algorithm is shown in Fig 2:

$$V_{\text{i}}^{Gen+1}=\omega V_{\text{i}}^{Gen}+c_{1}r_{1}\left(X_{i}^{pbest}-X_{i}^{Gen}\right)+c_{2}r_{2}\left(X_{gbest}-X_{i}^{Gen}\right)$$ (5)

$$X_{i}^{Gen+1}=X_{i}^{Gen}+V_{i}^{Gen+1}$$ (6)

${\text{c}}_{1}$ and ${\text{c}}_{2}$ are learning factors that respectively control individual and societal learning abilities; ${\text{V}}_{\text{i}}^{\text{Gen}+1}$ refers to the velocity of the i-th particle in Gen+1 generation. ${\text{X}}_{\text{i}}^{\text{pbest}}$ refers to the best position of the i-th particle so far. ${\text{X}}_{\text{gbest}}$ refers to the best position of all the particles. ${\text{X}}_{\text{i}}^{\text{Gen}+1}$ refers to the position of the i-th particle in Gen+1 generation. In the process of using PSO algorithm for emergency management and scheduling optimization of long-distance natural gas pipelines, the first step is to preliminarily configure the PSO algorithm. The initialization step determines the number of particle swarm and the size of each particle, and randomly generates initial positions within a reasonable range to ensure that the pipeline parameters corresponding to these positions meet the constraints of the pipeline network, such as pipeline flow rate and valve condition. The second step is to minimize the scheduling time and ensure that the pipeline scheduling scheme meets the constraints of the pipeline network through particle adaptive evaluation steps, and to construct an adaptive function. The adaptive function should balance scheduling time optimization and pipeline stability to improve the quality of the final solution. In the update stage of individual optimal position and overall optimal position, the individual optimal position and overall optimal position are updated based on the current particle position and fitness function value. During the search process, the optimal pipeline planning strategy found by each particle is represented by a single optimal position, while the optimal pipeline planning method in the particle swarm is represented by the overall optimal position. The updating process of particle velocity and position is based on inertia weight, individual learning factor, and social learning factor to update the velocity and position of particles. When updating, it is necessary to ensure that the position of the new particles meets the constraints of the pipeline network, such as pipeline flow restrictions, valve status restrictions, etc. Finally, in the iteration stage, after multiple iterations, the output optimal solution is obtained, and the particle swarm continues to iterate until the given number of iterations is reached or the stopping condition is met. Through iteration, a pipeline scheduling optimization scheme corresponding to the global optimal position, with the shortest scheduling time and meeting the constraints of the pipeline network, is ultimately obtained.

Although several new optimization algorithms have emerged in recent years (such as crawfish optimization, reptile search algorithm, and Red Fox optimization algorithm), PSO is still popular because it is simple to implement, has fewer parameters, and can be quickly applied and adjusted. It is also suitable for high-dimensional scheduling problems and performs stably in a variety of optimization scenarios, adapting to different constraints and objective functions. Notably, Wolpert’s No Free Lunch theorem states that no algorithm is universally superior on all optimization problems. Therefore, while PSO is outstanding in some scenarios, other new algorithms may be more effective on specific problems. The choice of PSO does not exclude the advantages of other methods, but rather emphasizes its effectiveness in a particular application.

### 4.2. Optimization of PSO algorithm

There are some complex problems in emergency management of long-distance natural gas pipelines. The long-distance natural gas pipeline network is usually composed of a large number of pipelines, each with multiple parameters such as length, diameter, flow rate, etc. This leads to a high-dimensional feature in the parameter space of the pipeline network [24]. High dimensional problems increase the computational complexity of optimization algorithms and make the search space [25] larger and more complex, making optimization problems more difficult to solve. Long-distance natural gas pipelines generally exhibit a large number of local minimum values [26], which may be due to the complexity of the pipeline network and various constraints. The pipeline network is influenced by various external factors, such as changes in weather conditions, human intervention, and pipeline aging. Under these complex conditions, the PSO algorithm cannot fully utilize its functionality in a large search space with numerous local hubs. Therefore, how to improve the diversity and convergence of PSO algorithms is a related research field. In response to the problem of PSO being prone to premature convergence [27] and relying on initial parameters, this article uses the Neighborhood Adaptive Constrained Fractional Particle Swarm Optimization (NAFPSO) [28,29] method. In the algorithm, the neighborhood topology is dynamically adjusted based on the evolutionary state to update particle positions and velocities, in order to improve the global optimization ability and convergence speed of feasible solutions; the penalty function constraint processing technique with penalty factors is adopted to force particles towards feasible regions; the differential mutation strategy is designed to increase population diversity and enhance the ability of particles to escape local optima.

The position update formula of the NAFPSO algorithm is shown in Formula (7):


$$x_{i}\left(t+1\right)=x_{i}\left(t\right)+v_{i}\left(t+1\right)$$ (7)

Among them, $x_{i}\left(t\right)$ is the position of particle *i* at time *t*, and $v_{i}\left(t+1\right)$ is the velocity of particle *i* at time t+1.

The velocity update of particles includes two parts: one is the inertia term, and the other is the guidance term for local and global information. The speed update formula of the NAFPSO algorithm is shown in Formula (8):


$$v_{i}\left(t+1\right)=w\cdot v_{i}\left(t\right)+c_{1}\cdot r_{1}\cdot \left(pbest_{i}\left(t\right)-x_{i}\left(t\right)\right)+c_{2}\cdot r_{2}\cdot \left(gbest\left(t\right)-x_{i}\left(t\right)\right)$$ (8)

In the formula, *w* is the inertia weight, which controls the degree of inertia of particles; $r_{1}$ and $r_{2}$ are random numbers; $pbest_{i}\left(t\right)$ is the individual optimal position of particle *i* at time *t*; $gbest\left(t\right)$ is the global optimal position.

**Fig 3:**
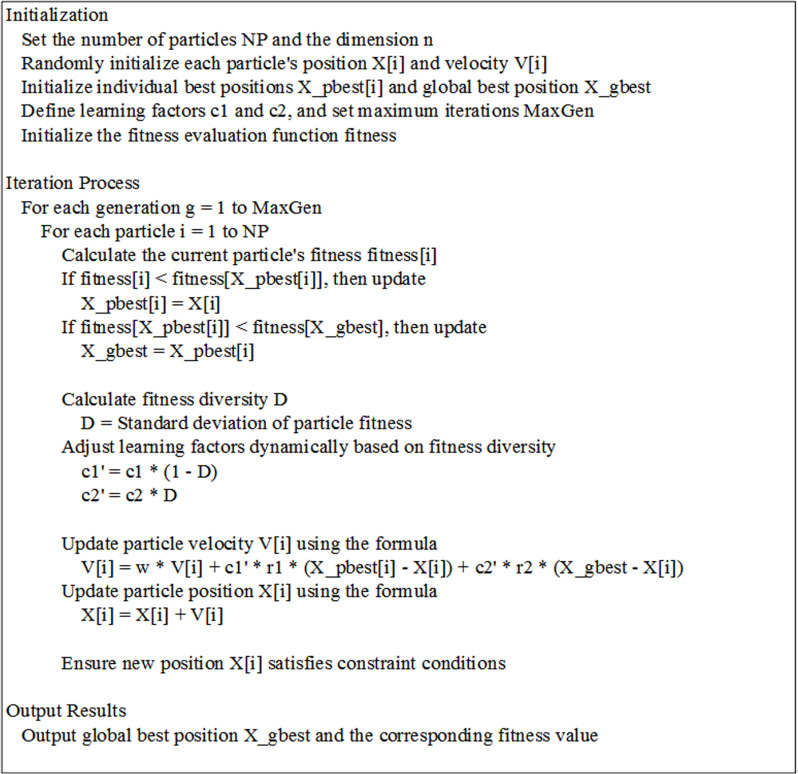
The pseudocode for the NAFPSO algorithm.

The NAFPSO algorithm also adopts an adaptive mechanism [30] to dynamically adjust the values of learning factors $c_{1}$ and $c_{2}$. The adaptive mechanism automatically adjusts the size of the learning factor based on the search progress and performance of the particle swarm to optimize the convergence speed and stability of the algorithm. In summary, the NAFPSO algorithm can effectively optimize the solution of the problem by updating the particle position and velocity, combined with fuzzy logic control and adaptive mechanism, and has certain optimization performance and robustness. The pseudocode for the NAFPSO algorithm is shown in Fig 3.

## 5. Simulation experiments

### 5.1. Experimental setup

#### Time and geographical scope.

The simulation experiment lasted for one week, totaling 168 hours. The simulation experiment covered the long-distance natural gas pipeline network of a certain city, including pipelines and related facilities.

#### Structure and parameters of pipeline networks.

The pipeline network consisted of 20 main pipelines and 80 nodes (including valves, connection points, etc.). The length (between 400 meters and 1000 meters), diameter (between 0.5 meters and 0.9 meter), and flow rate (between 50 cubic meters/hour and 100 cubic meters/hour) of each pipeline were randomly generated, as shown in Table 1:

**Table 1:** Structure and parameters of pipeline network.

Pipeline ID	Starting node	Intermediate node	Ending node	Length	Diameter	Flow rate
1	1	(21 24 27)	61	500	0.6	80
2	2	(22 25 28)	62	800	0.8	70
3	3	(30 33)	63	600	0.7	60
4	4	(21 34 37)	64	400	0.5	50
5	5	(23 26 29)	65	700	0.9	90
6	6	(32 35)	66	550	0.65	75
7	7	(36 39 42)	67	720	0.75	65
8	8	(38 41)	68	480	0.55	55
9	9	(40 43 46)	69	630	0.85	95
10	10	(44 47)	70	510	0.62	85
11	11	(45 48 51)	71	750	0.72	75
12	12	(49 52)	72	640	0.78	68
13	13	(50 53 56)	73	580	0.68	63
14	14	(54 57)	74	680	0.73	72
15	15	(55 58 21)	75	520	0.67	78
16	16	(22 59)	76	800	0.88	88
17	17	(23 26 29)	77	700	0.83	82
18	18	(25 28 31)	78	600	0.77	75
19	19	(24 27)	79	550	0.71	70
20	20	(30 32)	80	650	0.76	80

Note: The units of length and diameter are in meters, while the units of flow rate are in cubic meters per hour.

#### Probability and location of fault events.

It is assumed that the probability of each node and pipeline failing is evenly distributed at 0.1% over the course of a week. Fault location: The location of the fault event is randomly generated based on the probability distribution, and the fault details of the main fault [31] are shown in Table 2.

**Table 2:** Fault events.

Event ID	Type	Detection duration (hours)	Fault duration (hours)
1	Corrosion	24	10
2	Leakage	48	8
3	Mechanical Damage	72	15
4	Valve Failure	96	6
5	Pressure Abnormality	36	4
6	Equipment Failure	60	5
7	Pipeline Deformation	84	20
8	Gas Composition Abnormality	30	3

#### Resources and capabilities for emergency response.

There were 10 maintenance personnel available for emergency response, and 5 maintenance vehicles available for emergency response. There were three sets of maintenance equipment available for emergency response. The allocation of 10 maintenance personnel can ensure sufficient labor force to cope with pipeline failures of different scales; the setting of 5 maintenance vehicles can ensure rapid response and movement at the fault site; the provision of three sets of maintenance equipment can provide diverse tools and equipment to cope with different types of faults.

#### Parameter settings for PSO algorithm.

The inertia weight was set between 0.2 and 1.0, while the learning factor ranged from 1.0 to 3.0. The range of particle count was from 10 to 50. The maximum number of iterations was 100–600. The range of inertia weight and learning factor was set between 0.2 to 1.0 and 1.0 to 3.0, respectively. This setting can find a balance point between local search and global search, thereby improving the convergence speed and search ability of the algorithm. Through this flexible and diverse parameter setting, the PSO algorithm can adapt well to the characteristics of various problems, thereby improving the results and performance of scheduling optimization [32–34].

### 5.2. Performance evaluation and experimental results

Six sets of experiments with different parameters were designed to evaluate the convergence speed and scheduling time of PSO.

These parameters in the experiment had a significant impact on the velocity and position update rules of particles. The six sets of parameters for the experiment were set in these six sets of parameters: the number of particles was in the range of 10–35; the inertia weight was in the range of 0.5–0.9; the individual learning factor and social learning factor took the same value, with a value range of 1.5–2.5; the maximum number of iterations was between 100–350; the number of iterations was increasing. Because it is not a comparative experiment, the parameter setting in this study does not adopt the method of controlling variables. Instead, some parameters were incremented, and some parameters were randomly set within a certain interval, as long as the final convergence speed and scheduling time did not fluctuate too much. The specific parameters and results of the six experimental groups are shown in Tables 3 and 4:

**Table 3:** Experimental parameter combinations.

Parameter Set	N	Inertia	C1	C2	Max iterations	Pipeline complexity
Parameter Set1	10	0.5	1.5	1.5	100	0.5
Parameter Set2	20	0.7	2.0	2.0	200	0.5
Parameter Set3	30	0.9	2.5	2.5	300	0.5
Parameter Set4	15	0.6	2.2	2.2	150	0.5
ParameterSet5	25	0.8	1.8	1.8	250	0.5
Parameter Set6	35	0.9	2.3	2.3	350	0.5

Note: N, C1, and C2 in the table represent particle quantity, individual learning factor, and social learning factor, respectively.

**Table 4:** Experimental results.

	Parameter Set1	Parameter Set2	Parameter Set3	Parameter Set4	Parameter Set5	Parameter Set6
Convergence speed (in iterations)	82	83	81	84	80	85
Scheduling time (minutes)	205	208	203	206	200	210

To ensure the operability of experimental data, it is necessary to ensure that the complexity of the pipeline is consistent, and to obtain the changes in convergence speed and scheduling time under different particle numbers. In this data, there was no significant trend in convergence speed and scheduling time as the number of particles increased. The convergence speed was in the range of 80–85 iterations, and the scheduling time was in the range of 200–210 minutes. When the number of particles was 25, the inertia weight was 0.8, and the learning factor was 1.8. When the maximum number of iterations was 250, the convergence speed and scheduling time were optimal. The convergence speed was 80 iterations, and the scheduling time was 200 minutes.

**Fig 4:**
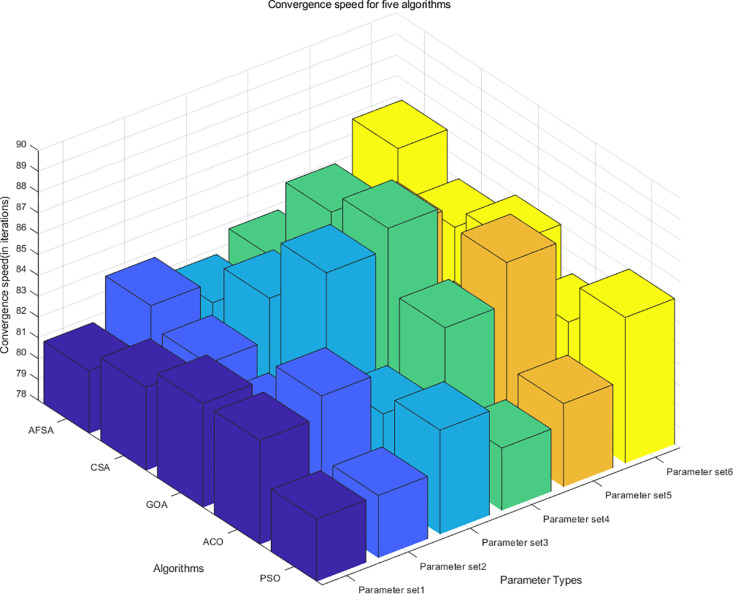
Convergence speed of five algorithms.

**Fig 5:**
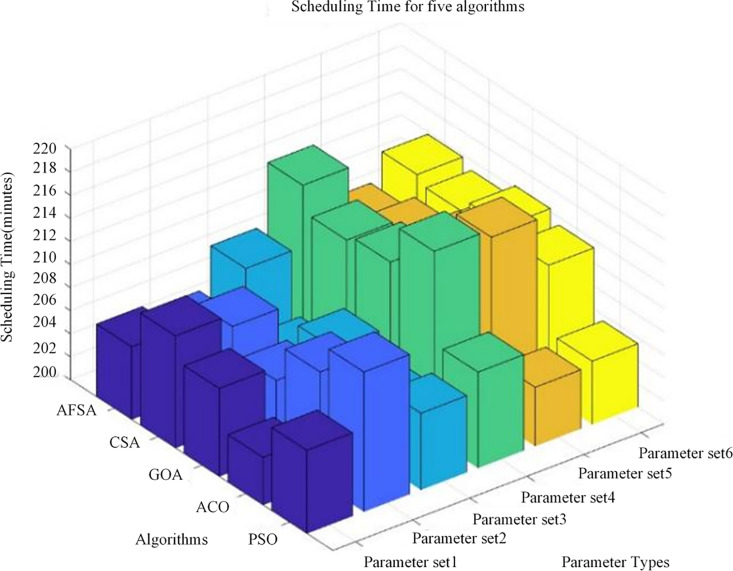
Scheduling time of five algorithms.

However, this experiment can only demonstrate the convergence speed and minimum scheduling time of the PSO algorithm in emergency management of long-distance natural gas pipelines, and cannot demonstrate the advantages of PSO in minimizing scheduling time [35]. Therefore, in order to fully verify the superiority of the optimization scheduling model established in this chapter and the effectiveness of the PSO algorithm, the experimental results of PSO, AFSA, CSA, GOA, and ACO algorithms were compared under the same simulation conditions. In order to eliminate randomness, each group of experiments was independently repeated 30 times, and the mean of 30 experiments was taken. The experimental results are shown in Figs 4 and 5:

Figs 4 and 5 show the convergence speed and scheduling time of five algorithms in emergency management of long-distance natural gas pipelines under six different parameters. The differences in convergence speed and scheduling time among these five algorithms were relatively small, so the approach of taking the mean and then retaining two decimal places was adopted. The convergence speed range of AFSA algorithm was 81–86 iterations (mean 83.00), and the scheduling time range was 205–215 minutes (mean 209.55); the convergence speed range of CSA algorithm was 82–87 iterations (mean 84.00), and the scheduling time range was 204–213 minutes (mean 209.45); the convergence speed range of the GOA algorithm was 81–88 iterations (mean 84.33), and the scheduling time range was 206–213 minutes (mean 209.76); the convergence speed range of the ACO algorithm was 82–87 iterations (84.00), and the scheduling time range was 204–217 minutes (mean 210.47); the convergence time and scheduling time of the PSO algorithm used in this article were 81–85 iterations (mean 82.17) and 205–213 minutes (mean 207.49), respectively. In terms of convergence speed and scheduling time, the PSO algorithm was superior to the other four algorithms, proving the feasibility and applicability of the algorithm.

Due to the randomness of the meta-heuristic algorithm, the PSO should be run independently several times in the same setting. On the basis of these independent operations, the statistical results of the total scheduling time of the objective function of the PSO algorithm were recorded, as shown in Table 5.

**Table 5:** The statistics of the total time of the objective function scheduling.

Run times	Optimum value (T_total)	Worst value (T_total)	Average value (T_total)	Median (T_total)	Standard deviation	Variance
1	195.2	205.3	199.4	198.5	3.6	12.96
2	192.8	207.1	198.1	197.0	4.1	16.81
3	193.5	206.0	199.0	198.2	3.8	14.44
4	190.9	204.5	197.6	196.0	4.0	16.00
5	194.3	208.0	199.3	198.1	3.7	13.69

### 5.3. NAFPSO simulation experiment

The optimized mathematical model still used most of the data from the PSO experiment mentioned earlier to facilitate comparison with the PSO algorithm. The only difference was the complexity of long-distance natural gas pipelines, which was uniformly set at 0.5 (the complexity was determined by various parameters of the pipeline, such as length, diameter, flow rate, etc.) [36–38]. This experiment set three complexity levels, namely 0.5, 0.6, and 0.7. The experimental results are shown in Tables 6–8.

**Table 6:** Experimental results of NAFPSO algorithm with pipeline complexity of 0.5.

Parameter Set	Pipeline complexity	Convergence speed (in iterations)	Scheduling time (minutes)
Parameter Set1	0.5	82	197
Parameter Set2	0.5	79	202
Parameter Set3	0.5	82	195
Parameter Set4	0.5	83	201
Parameter Set5	0.5	83	200
Parameter Set6	0.5	78	205

**Table 7:** Experimental results of NAFPSO algorithm with pipeline complexity of 0.6.

Parameter Set	Pipeline complexity	Convergence speed (in iterations)	Scheduling time (minutes)
Parameter Set1	0.6	80	202
Parameter Set2	0.6	84	198
Parameter Set3	0.6	79	204
Parameter Set4	0.6	85	200
Parameter Set5	0.6	83	196
Parameter Set6	0.6	78	206

**Table 8:** Experimental results of NAFPSO algorithm with pipeline complexity of 0.7.

Parameter Set	Pipeline complexity	Convergence speed (in iterations)	Scheduling time (minutes)
Parameter Set1	0.7	82	198
Parameter Set2	0.7	84	202
Parameter Set3	0.7	81	198
Parameter Set4	0.7	85	205
Parameter Set5	0.7	83	199
Parameter Set6	0.7	80	206

The purpose of setting the complexity to 0.5 in Table 6 is to compare it with the PSO algorithm mentioned earlier. From the data in Table 6, it can be seen that the convergence speed of NAFPSO was 78–83 iterations, with a scheduling time of 195–205 minutes. In PSO, with the same pipeline complexity of 0.5, the convergence speed was 81–85 iterations, with a scheduling time of 205–213 minutes. When the pipeline complexity was 0.5, the convergence speed and scheduling time of the NAFPSO algorithm were better than those of the PSO algorithm. However, observing the evaluation range formed by the six sets of parameters cannot accurately measure the superiority or inferiority of the algorithm, as the final result may be unevenly distributed. Therefore, in order to solve this problem, the average of six sets of parameter results was used as a comparison of the final effect.

As shown in Table 9, when the pipeline complexity was 0.5, the average convergence speed of NAFPSO was 81.17 iterations, and the average scheduling time was 200.00 minutes. However, when the pipeline complexity was 0.5, the average convergence speed of PSO was 82.17 iterations, and the average scheduling time was 207.49 minutes. When the pipeline complexity was 0.5, both the average convergence speed and average scheduling time of NAFPSO were better than PSO algorithm. The pipeline complexities set in Tables 7 and 8 were 0.6 and 0.7, respectively. When the complexity increases, if it can still outperform or be on par with the convergence speed and scheduling time of PSO at a complexity of 0.5, then the NAFPSO algorithm can be considered a successful optimization. The experimental results showed that when the complexity was 0.6, the average convergence speed and average scheduling time of NAFPSO were 81.50 iterations and 201.00 minutes, respectively. At this time, the two evaluation indicators of the algorithm NAFPSO were still better than PSO. When the pipeline complexity was 0.7, the average convergence speed of the two algorithms was not significantly different, but the scheduling time of NAFPSO was still much better than PSO, further verifying the superiority of NAFPSO. The t-test was used for statistical analysis of the obtained results. Results are shown in the Table 10.

**Table 9:** Average convergence speed and average scheduling time of NAFPSO under three pipeline complexities.

Pipeline complexity	Average convergence speed (in iterations)	Average scheduling time (minutes)
0.5	81.17	200.00
0.6	81.50	201.00
0.7	82.50	201.33

**Table 10:** Statistical test of experimental results of NAFPSO and PSO algorithm.

Pipeline complexity	Algorithm	Average convergence speed (in iterations)	Standard deviation (velocity)	Average scheduling time (minutes)	Standard deviation (time)	t-statistic	P value
0.5	NAFPSO	81.17	2.16	200.00	3.73	−2.28	0.03
	PSO	82.17	1.63	207.49	3.84		

In Table 10, the t-test results showed that the P-value was 0.03, indicating that NAFPSO had significant advantages in convergence speed and scheduling time.

## 6. Conclusions

In order to solve the problems of poor control, inefficient resource utilization, and especially lack of effective resource utilization faced by natural gas pipeline scheduling methods, this paper applies the scheduling strategy of PSO to multi-constraint problems, and further proposes a NAFPSO algorithm. By dynamically adjusting the neighborhood topology structure and applying penalty function constraint processing technology, the diversity and convergence speed of the algorithm are improved. This paper verifies the superior performance of the NAFPSO algorithm in natural gas pipeline emergency management scheduling through simulation experiments. Compared with the PSO algorithm, NAFPSO has advantages in convergence speed and scheduling time. Of course, this paper also has certain shortcomings, and the parameter setting still needs to be further optimized and adjusted. Future research can build a hybrid optimization model and an intelligent decision support system on this basis to further improve the emergency management efficiency and response capability of the natural gas pipeline network, and provide strong support and guidance for future pipeline scheduling decisions.

## Supporting information

S1 Data Data sources for figures and tables. (ZIP)
